# Supplementary material for: Fluid Shear Stress-Induced Changes in Megalin Trafficking Enhance Endocytic Capacity in Proximal Tubule Cells
Source: bioRxiv. 2024 Mar 19:2024.02.22.581213. Preprint. [Version 2] doi: 10.1101/2024.02.22.581213 (PMC10983855; doi:10.1101/2024.02.22.581213)

Supplemental Materials

Table S1. Antibodies for indirect immunofluorescence in OK cells

Primary Antibody Target or Secondary	Company	Catalog #	Source	Working Dilution
Megalin	(Zou et al. 2004)	--	Rabbit	1:1000
Rab11a	Abcam	ab65200	Rabbit	1:500
Rab7	Sigma	R8779	Mouse	1:200
Cathepsin B	Cell Signaling	31718	Rabbit	1:500
EEA1 (E-8)	Santa Cruz	sc-365652	Mouse	1:50
Anti-mouse Alexa Fluor 488	Invitrogen	A11029	Goat	1:500
Anti-rabbit Alexa Fluor 488	Invitrogen	A11034	Goat	1:500
Anti-mouse Alexa Fluor 647	Invitrogen	A21236	Goat	1:500
Anti-mouse Alexa Fluor 647	Invitrogen	A21245	Goat	1:500
F(ab) anti-mouse Alexa Fluor 488	Jackson Immuno	115-547-003	Goat	1:500
F(ab) anti-rabbit Alexa Fluor 488	Jackson Immuno	111-547-003	Goat	1:500
F(ab) anti-mouse (unconjugated)	Jackson Immuno	115-007-003	Goat	1:65
F(ab) anti-rabbit (unconjugated)	Jackson Immuno	111-007-003	Goat	1:65

Table S2. Average fractional colocalization (Manders' coefficient) of megalin with endocytic markers

	static			FSS		
**Manders' Coefficient**	**Mean**	**SEM**	**N**	**Mean**	**SEM**	**N**
M_{EEA1}	0.0975	0.0216	24	0.1287	0.0238	40
M_{Rab7}	0.2533	0.0253	24	0.2979	0.0218	39
M_{Rab11}	0.5731	0.0454	20	0.5824	0.0255	30
M_{CTSB}	0.0213	0.0040	15	0.0807	0.0160	15

Table S3. Average fractional overlap (Manders' coefficient) between endocytic markers

	static			FSS		
**Manders' Coefficient**	**Mean**	**SEM**	**N**	**Mean**	**SEM**	**N**
$EEA1_{Rab7}$	0.5073	0.0555	29	0.5583	0.0436	49
$EEA1_{Rab11}$	0.2539	0.0194	20	0.2970	0.0197	35
$Rab7_{EEA1}$	0.1587	0.0235	29	0.2513	0.0253	49
$Rab7_{Rab11}$	0.2602	0.0429	25	0.4466	0.0333	35
$Rab7_{CTSB}$	0.0180	0.0026	10	0.0217	0.0048	10
$Rab11_{EEA1}$	0.0663	0.0077	20	0.0792	0.0191	35
$Rab11_{Rab7}$	0.1415	0.0248	25	0.2528	0.0260	35
$Rab11_{CTSB}$	0.0164	0.0054	10	0.0193	0.0088	9
$CTSB_{Rab7}$	0.3487	0.0297	10	0.0633	0.0093	10
$CTSB_{Rab11}$	0.4652	0.0534	10	0.2443	0.0563	9

Supplemental Figure Legends

Figure S1. Quantification of cell heights in static- and FSS-cultured cells. (A) OK cells on permeable supports were fixed and stained for megalin, Rab11a and actin. To determine cell height, using ImageJ software and XZ stacks, line objects were drawn from the base of the cell to the tip of the microvilli based on actin staining. Each dot represents the height of one cell. ****P=<0.0001 by unpaired t-test, n=85-86 cells. (B-C) Representative images of actin, megalin, and Rab11 staining in static-cultured (B) and FSS-cultured (C) cells. XZ images are sum projections of the entire cell stack. Scale bars: 2μm.

Figure S2. Quantification of the overlap between endocytic markers in static- and FSS-cultured cells. (A-C) OK cells on permeable supports were fixed and processed to detect colocalization between the indicated pairs of endocytic compartment markers. Quantification by Manders' coefficient over the entire z-stack, shown as the % of total for the following: (A) EEA1 and Rab7, (B) Rab7 and Rab11, and (C) EEA1 and Rab11. Each point represents a single z-stack image. *P=0.0345, ***P=0.0002 by Two-way ANOVA with multiple comparisons.

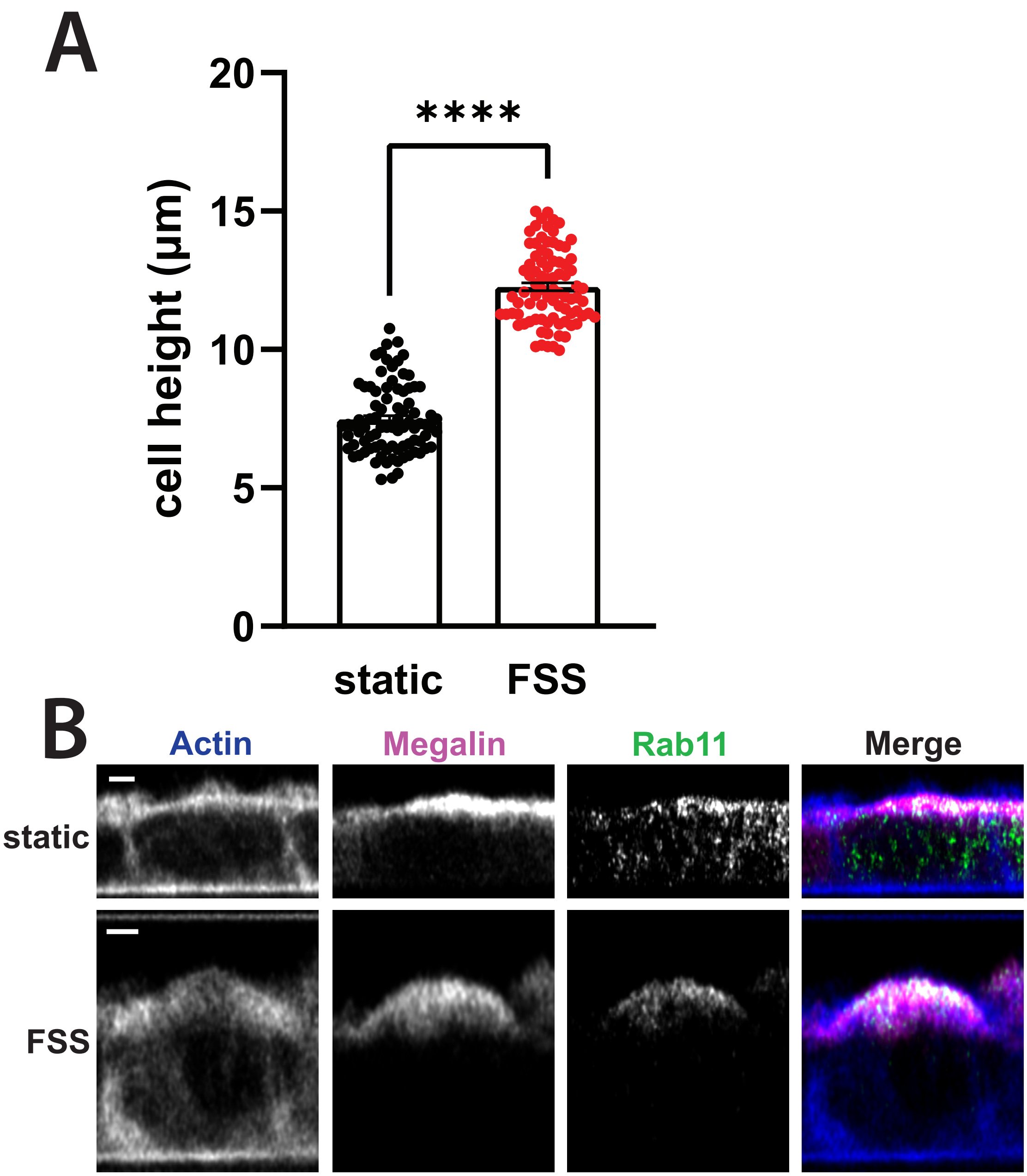
A
20
15
10
5
0
cell height (μm)
****
static
FSS
B
Actin
Megalin
Rab11
Merge
static
FSS

Lackner et al. Figure S2

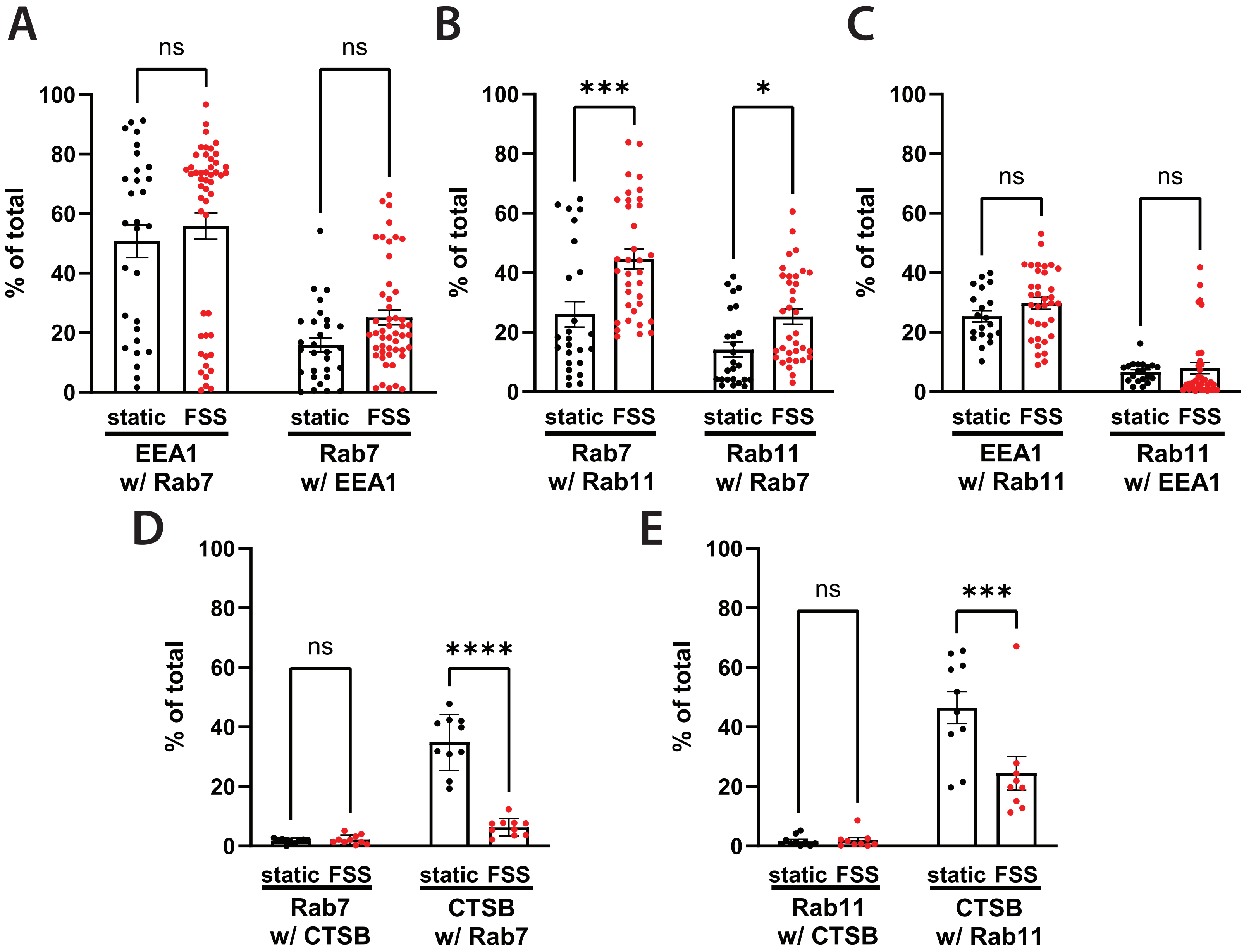

Supplement: Supplement 1 [file media-1.pdf]
